# Cell Pair Algorithm-Based Immune Infiltrating Cell Signature for Improving Outcomes and Treatment Responses in Patients with Hepatocellular Carcinoma

**DOI:** 10.3390/cells12010202

**Published:** 2023-01-03

**Authors:** Xiao Zhang, Jun Xie, Dan He, Xin Yan, Jian Chen

**Affiliations:** 1 Department of General Surgery, Hospital of Chengdu Office of People’s Government of Tibet Autonomous Region, Chengdu 610041, China; 2 The Second Clinical College, Zhongnan Hospital of Wuhan University, Wuhan 430071, China; 3 Department of Gastrointestinal Surgery, Zhongshan Hospital of Xiamen University, Xiamen 361004, China; 4 Department of Urology, Zhongnan Hospital of Wuhan University, Wuhan 430071, China; 5 Department of Emergency Department, The Fourth Affiliated Hospital of Zhejiang University School of Medicine, Yiwu 322000, China

**Keywords:** immune cell, immunotherapy, hepatocellular carcinoma, prognosis, treatment, tumor biomarkers

## Abstract

Background: Immune interactions play important roles in the regulation of T cells’ cytotoxic function, further impacting the anti-tumor efficacy of immunotherapy. A comprehensive analysis of immune cell types in HCC and immune-cell-related signatures predicting prognosis and monitoring immunotherapy efficacy is still absent. Methods: More than 1,300 hepatocellular carcinomas (HCC) patients were collected from public databases and included in the present study. The ssGSEA algorithm was applied to calculate the infiltration level of 28 immunocyte subpopulations. A cell pair algorithm was applied to construct an immune-cell-related prognostic index (ICRPI). Survival analyses were performed to measure the survival difference across ICRPI risk groups. Spearman’s correlation analyses were used for the relevance assessment. A Wilcoxon test was used to measure the expression level’s differences. Results: In this study, 28 immune subpopulations were retrieved, and 374 immune cell pairs (ICPs) were established, 38 of which were picked out by the least absolute shrinkage and selection operator (LASSO) algorithm. By using the selected ICPs, the ICRPI was constructed and validated to play crucial roles in survival stratification and dynamic monitoring of immunotherapy effect. We also explored several candidate drugs targeting ICRPI. A composite ICRPI and clinical prognostic index (ICPI) was then constructed, which achieved a more accurate estimation of HCC’s survival and is a better choice for prognosis predictions in HCC. Conclusions: In conclusion, we constructed and validated ICRPI based on the cell pair algorithm in this study, which might provide some novel insights for increasing the survival estimation and clinical response to immune therapy for individual HCC patients and contribute to the personalized precision immunotherapy strategy of HCC.

## 1. Introduction

As the sixth most commonly diagnosed cancer and the third leading cause of cancer death worldwide in 2020, liver cancer has a high incidence rate and mortality [1]. About 75% of the new primary liver cancer patients in the world each year exhibit hepatocellular carcinoma (HCC). As the global cancer statistics in 2020 reported, it was estimated that 905,677 new cases and 830,180 new deaths would be reported in 2020 [2]. As the American Cancer Society reported, there will be about 41,260 new liver cancer cases and about 30,520 new deaths in the United States in 2022 [3]. According to the statistics provided by the American Society of Clinical Oncology (ASCO, www.cancer.net/ accessed on 1 October 2022), the 5-year survival rate of HCC is 20%, but this figure was only 3% 40 years ago. The survival rate of patients is usually affected by a set of features, and the disease stage is one of the major factors. When HCC is diagnosed in the early stage, it has a 34% 5-year survival rate. However, when HCC spread to the surrounding tissues/regional lymph nodes, the rate drops sharply to 12%. Even worse, when HCC metastasizes far away, the rate is only 3%. Nevertheless, even if HCC is discovered at a very advanced stage, therapies can effectively prolong life cycles and enhance the quality of life [4]. If surgery works, it usually leads to a higher survival rate for patients at all stages.

As a type of cancer treatment, immunotherapies treat cancer by helping the immune system defend against cancer [5]. These days, there is a growing realization that immunotherapy for cancers is effective and secure [6,7,8]. Exploring immune-related prognostic biomarkers has been one of the ineluctable research highlights in tumor therapy. Over the past 5 years, immune-checkpoint inhibitors have brought revolutionary changes to the management of HCC. The combination of atezumab and bevacizumab in the treatment of HCC was currently approved by FDA because it could improve the overall survival rate compared with sorafenib [9]. Recent research data show that the overall survival rate of duvalimab combined with trimetazumab was better than that of sorafenib, while the progression-free survival rate of atizumab combined with cabozadinib was higher than that of sorafenib (https://www.astrazeneca.com/media-centre/ accessed on 1 October 2022). Despite these significant advances, the molecular underpinnings governing immune responses and escape remain unclear [10]. The immune microenvironment (IME) is an essential part of the development and progression of HCC, and it defined different etiologically dependent immune characteristics [11]. As the important components of IME, immune cells (including T cells, B cells, macrophages, etc.) represent the major components of antitumor immune reaction [12,13]. Recent research studies have shown that immune cells are correlated with the prognosis of cancers, which could also accelerate tumor initiation and progression [14,15,16]. However, to the best of our knowledge, studies concentrating on immune cell subpopulations are still lacking, which indicates that a deep understanding of the activity of immune cells in the tumor microenvironment (TME) is still required.

Charoentong et al. firstly developed an immune cell infiltration-level estimation method with the use of metagenes [17], which has been broadly used in the field of immunity. Two studies contributed by Wang et al. published in Briefings in *Bioinformatics* successfully applied this method in breast cancer [18,19]. The immune cell signature could act as a prognostic index in breast cancer. Moreover, Ye et al. proved that tumor-infiltrating immune cells could act as markers for prognosis in colorectal cancer [20]. Thus, we thought it was also feasible to construct a prognostic index based on immune cells in HCC. Studies only based on a single dataset or method cannot draw a good conclusion. So, many studies have begun to conduct analysis using multiple datasets to improve the feasibility of their conclusions [21]. However, due to the differences between the abilities of researchers to process data, the conclusions of the studies are sometimes unreliable. To overcome the difficulty of using multiple datasets from different platforms, we have reviewed several studies. A paper from *JAMA Oncology* contributed by Li et al. has attracted our attention [22]. They used methods based on the relative ranking of gene expression levels to construct a prognostic signature in early-stage nonsquamous non-small-cell lung cancer, which could effectively eliminate the potential biological heterogeneity among data cohorts and technical biases across measurement platforms and produce robust results in various applications. Based on this, Zhang et al. constructed an immune infiltrating cell signature using a method based on a relative ranking of immune cell infiltration levels―immune cell pair (ICP)―which was validated as prognostic index in glioma [23]. Moreover, Yan et al. constructed a novel T-cell signature based on the cell pair algorithm, which could predict survival and immunotherapy response for bladder cancer patients [24].

Thus, in this research paper, for the first time, via the utilization of multiple meta-HCC cohorts, we first measured the immune cell infiltration level of each HCC among these cohorts. Then, an immune cell pair (ICP) was constructed by using the 28 immune cell types with the aim of simulating immune cell interactions. Prognostic ICPs were screened and used to construct an immune-cell-related prognostic index (ICRPI) by acting on prognosis predication and immunotherapy efficacy monitoring. 

## 2. Methods

### 2.1. Collection of HCC Cohorts and the Related Clinical Characterization

The HCC expression cohorts were retrieved from several public databases, including ArrayExpress (*n* = 1), gene expression omnibus (GEO) database (*n* = 7), the International Cancer Genome Consortium (ICGC) database (*n* = 1), and the Cancer Genome Atlas (TCGA) database (*n* = 1) (Table S1). Only HCC cohorts with more than 40 HCC samples and related survival and clinical information were collected and used in the present study. A total of 10 HCC cohorts with survival information were included in our study. The related literature of these cohorts is also shown in Table S1. TCGA-LIHC RNA-seq data were firstly downloaded for the TCGA database. R package “DEseq.2” [25] was used for normalization and log2 transformation. For the GEO cohorts platformed on Affymetrix, raw CEL files were retrieved. We applied the robust multichip average (RMA) algorithm for normalization via R package “affy” [26]. For the GEO cohorts platformed on Illumina, we directly retrieved the normalized expression profiles for the GEO database. Package “sva” [27] in R software was used to merge the entire meta-cohort via the 10 collected cohorts using the following three steps: data preprocessing; merging; and ComBat-adjusted handling. In total, 717 normal samples and 1340 HCC samples were collected. The expression matrix of the 717 normal samples was only used for batch normalization, which was not included in the subsequent analysis. In the present study, 1340 HCCs containing complete survival information were used. To make sure whether the cohort of the patients was sufficient, we also performed power calculation via R package “pwr” in this study. As Figure S1 shows, in all cases (*t*-test: Figure S1A,B; one-way ANOVA: Figure S1C; correlation analysis: Figure S1D), the cohort of the patients was sufficient.

### 2.2. Application of Cell Pair Algorithm to Construct IMMUNE-Cell-Related Prognostic Index (ICRPI)

Charoentong et al. developed an analytical strategy for characterizing the cellular composition of immune infiltrates based on the use of metagenes [17]. They defined a set of pan-cancer metagenes for 28 immune cell subpopulations, which has been widely used for immune cell infiltration level estimations. Hence, this metagene-based method was used in the present study to measure the immune cell infiltration level in every HCC based on a single-sample gene set enrichment analysis (ssGSEA) realized by R package “GSVA” [28]. With the aim of avoiding differences among different data cohorts and improving the use of multiple cohorts, the immune-cell-related prognostic index (ICRPI) was constructed by using cell pair algorithms. Specifically, an immune cell pair score (ICPs) was defined as 1 when the infiltration level of immune cell subpopulation 1 was more than the infiltration level of immune cell subpopulation 2. Otherwise, the ICPs were assigned 0 (the infiltration level of immune cell subpopulation 1 was less than the infiltration level of immune cell subpopulation 2). Some ICPs with constant values (0 or 1) were removed for analyses in the next step to minimize the biases caused by the platform-dependent preferential measurement. We immediately contained these ICPs for identifying prognostic ICPs by using survival analyses (log-rank test method) based on the entire meta-cohort. The entire meta-cohort was randomly classified into a meta-training cohort and meta-testing cohort with a ratio of 1:1. By using package “glmnet” [29] in R software, we conducted a least absolute shrinkage and selection operator (LASSO) penalized Cox regression analysis of the selected prognostic ICPs via the meta-training cohort. The coefficients of the ICPs in the multivariate Cox proportional hazards model were contained for ICRPI construction. The ICRPI score of each HCC patient was evaluated by using Formula (1).

(1) $$\mathrm{ICRPI}=\sum_{i=1}^{n}Coef_{i}\times TCPs_{i}$$

Formula (1). The calculation formula of the immune-cell-related prognostic index (ICRPI). Coef represents the regression coefficient, and ICPs represent the immune cell pair score of each immune cell pair (ICP).

Here, Coef represents the regression coefficient, and ICPs represent the immune cell pair score of each ICP (immune cell pair). To divide HCC patients into high- and low-risk groups appropriately, a time-dependent receiver operating characteristic (ROC) curve was plotted. This analysis was performed based on the training cohort with the use of the “survivalROC” [30] package. The time point for this analysis was set as “5 years”, and then the ICP showing the minimum distance between the ROC curve and the point was further determined as the grouping cutoff value in the present study. HCC patients across the meta-training cohort, meta-testing cohort, entire meta-cohort, and TCGA-LIHC cohort were separated into a high-risk group and a low-risk group (ICRPI risk groups). 

### 2.3. Correlation between ICRPI and Survival, Clinical Characteristics, Pathological feaTures, and the Genomic Alterations of HCC Patients

To explore the prognostic value of the ICRPI, we then conducted survival analyses by using R package “survival”. The survival difference between the ICRPI risk groups were measured via the two cohorts. The prognostic role of the ICRPI was also validated via the entire meta-cohort and TCGA-LIHC data. A log-rank test was chosen for measuring the survival difference, with statistical significance when the *p* value was <0.05. Furthermore, we attempted to explore the clinical difference between low- and high-risk groups. Several clinical indicators containing age, gender, and TNM stage were contained for exploring clinical differences. Because of the importance of pathological features in HCC, pathologic M, pathologic N, and pathologic T were collected from TCGA data to explore the relation of ICRPI to pathological features. Moreover, metastasis, intrahepatic metastasis, and vascular invasion were collected from cohort GSE45114 to explore pathological differences. We performed Fisher’s exact test for determining the statistical differences among the groups, with statistical significance when the *p* value was < 0.05. The single-nucleotide variant (SNV) data of LIHC containing 367 samples were also obtained from the TCGA database. The mutation landscape in HCC patients grouped by ICRPI was presented by using R package “maftools” [31].

### 2.4. Association between ICRPI and Several Mutation and Immune Indices

Microsatellite instability (MSI) occurs because of functional defects in DNA mismatched repairs in neoplastic tissue. MSI accompanied by DNA mismatch repair defects is a noteworthy marker of malignancy in clinics [32]. For the present study, we calculated the MSI for each sample from the TCGA-LIHC cohort via R package “PreMSIm” [33]. The mRNA stemness index (mRNAsi) has been identified as a novel predictor that is associated with stem-like indices and tumor prognoses [34]. Thus, we collected mRNAsi, mDNAsi, EREG-mDNAsi, and EREG-mRNAsi from a previous research study and further explored the difference among the ICRPI groups. Homologous recombination deficiency (HRD) scores represent distinct types of genomics scar and chromosomal instability caused by deoxyribonucleic acid repair deficiency and, thus, are regarded as powerful biomarkers of a given cancer [35]. Thus, the HRD scores for the ICRPI risk groups were calculated to compare their chromosomal instability with Wilcoxon test. The index of cytolytic activity (CYT) is measured as a new biomarker of immunotherapy, which could characterize the antitumor immunity of CD8 + cytotoxic T cells and macrophages. We then evaluated the CYT score for each sample across the TCGA-LIHC data; in detail, the CYT score was defined as the mean expression of PRF1 and GZMA [36]. Cohort GSE104580, including 147 HCC patients with transcatheter arterial chemoembolization (TACE) treatment information, was obtained from the GEO database for the exploration of the predictive potential of ICRPI. The normalization of this cohort was conducted as described above. Then, the difference between the ICRPI risk groups were also measured by using the Wilcoxon test. A *p* value of <0.5 was considered significant.

### 2.5. Association between ICRPI and Immunotherapy-Predicted Pathways

Some therapeutic signatures containing oncogenic pathways that might form non-inflammatory TME, gene characteristics related to targeted therapy, and gene signatures for radiotherapy response prediction were also retrieved from Mariathasan’s study and Hu’s study [37,38]. Then, we explored the association between ICRPI and immunotherapy-predicted pathways. Pearson’s correlation analyses were performed, and a *p* value < 0.05 was considered significant.

### 2.6. Association between ICRPI and Immune-Related Features

To further explore the potential functions of ICRPI and provide an immune landscape for ICRPI, we calculated the immune score, stromal score, and tumor purity of HCCs via R package “ESTIMATE” [39] by using TCGA data. The T cell dysfunction and exclusion (TIDE) method was used for observing the response of immunity treatments and it was also quantified for HCC samples. The TIDE scores of the HCC samples from the TCGA-LIHC cohort were evaluated and retrieved via the following website: http://tide.dfci.harvard.edu/ accessed on 1 October 2022. Furthermore, by using a pan-cancer analysis of immune subtypes, Thorsson et al. defined 6 immune-related subtypes including wound healing (C1), IFN-γ-dominant (C2), inflammatory (C3), lymphocyte-depleted (C4), immunologically quiet (C5), and TGF-β-Dominant (C6) [40]. We attempted to explore the differences between these subtypes and the ICRPI. The authors in this study also defined 56 molecular signatures associated with immune characteristics [40]. Thus, we measured the correlation of ICRPI with these signatures. In view of the major significance of immune checkpoints (ICPs) and immunogenic cell death (ICD) modulators for tumor immunity, the associations between ICRPI with ICPs and ICD modulators were explored. The Wilcoxon method was used for statistical examinations. We also measured 28 immune cell components among the ICRPI risk groups.

### 2.7. Exploring the Role of ICRPI in Response to Anti-PD-1/L1 Immunotherapy

We obtained 2 immunotherapy-related datasets containing a gene expression matrix and survival information for the potential exploration of treatment responses. The expression matrix of cohort IMvigor210, patients of which were treated by atezolizumab (an anti-PD-L1 antibody), was firstly normalized via the method described previously. In total, we contained 298 cancer patients for this step analysis. Moreover, the expression matrix of cohort GSE78220, patients of which were treated by pembrolizumab (an anti-PD-1 antibody), was further downloaded via the GEO website. Normalization was performed via package “limma” [41]. Then, 27 cancer patients with their immunotherapy and survival information were contained for this analysis. We firstly explored the survival difference across the ICRPI risk groups via R package “survival”. Secondly, the ICRPI difference between different response groups (CR, PR, PD, and SD) was explored and the Kruskal–Wallis test was chosen to test the significance. Thirdly, by using R package “pROC” [42], receiver operating characteristic (ROC) curves were plotted to measure the prediction values of ICRPI for immunotherapy responses. The prediction potential was quantified by the area under the curve (AUC).

### 2.8. Construction and Verification of a Composite ICRPI and Clinical Prognostic Index (ICPI)

To obtain the prediction value of ICRPI and compare the prognostic accuracy of ICRPI with other HCC-related signatures, we collected 3 existing molecular signatures including a 3-gene signature [43], 6-gene signature [44], and 9-gene signature [45]. For these signatures, concordance indices (C-index) were calculated and considered as the comparison standards. Moreover, ICRPI and several clinical indicators (gender, age, and TNM stage) were included for multivariable Cox proportional regression analyses by using the four cohorts. Then, features showing significant values (*p* < 0.05) were used to establish the composite ICRPI and clinical prognostic index (ICPI). Similarly, we also evaluated the C-index of the ICPI via the four cohorts. In addition, the prognostic performances of continuous ICRPI and ICPI scores were compared by taking the C-index as the standard. The restricted mean survival (RMS) curve was used for the visualization of a continuous C-index. RMS represents the life expectancy at 10 years for patients with different indicators [46]. The higher the RMS time ratio, the greater the prognostic potential.

### 2.9. Drug Sensitivity Exploring

Then, we attempted to identify several novel therapeutic drugs, which could provide novel choices for HCC treatments. Based on the related drug information provided by the Genomics of Drug Sensitivity in Cancer (GDSC) database [47], we predicted the drug response in HCC with the use of package “pRRophetic” [48]. Ridge’s regression was firstly used to estimate the maximum inhibitory concentration (IC_50_) of each patient. Then, a 10-fold cross-validation was used to measure the accuracy of the estimation. We further divided the patients into high- and low-risk groups according to the level of ICRPI, and the Wilcoxon rank-sum test was used to measure the significance; *p* < 0.05 was considered significant.

## 3. Results

### 3.1. ICRPI Construction

**Figure 1:**
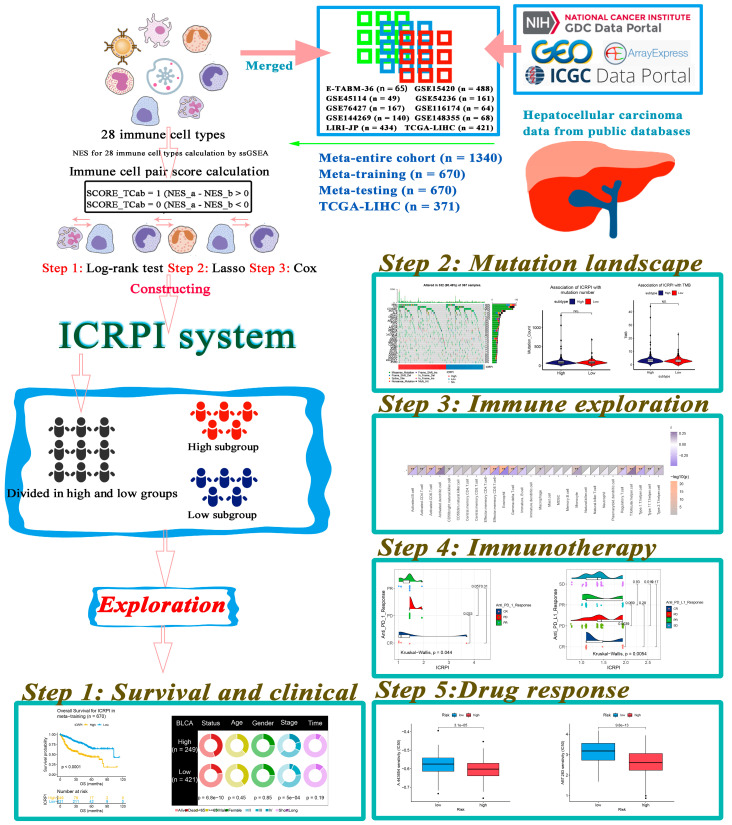
The flow diagram of this study. Data preparation, analysis, and validation are shown in the flow diagram. In total, 1340 HCC patients were included in this study, and the immune-cell-related prognostic index (ICRPI) was constructed by using cell pair algorithms, log-rank test, Lasso, and Cox analysis. Then, we explored the role of ICRPI in survival and clinical conditions, mutation landscape, immune exploration, immunotherapy, and drug therapy.

**Figure 2:**
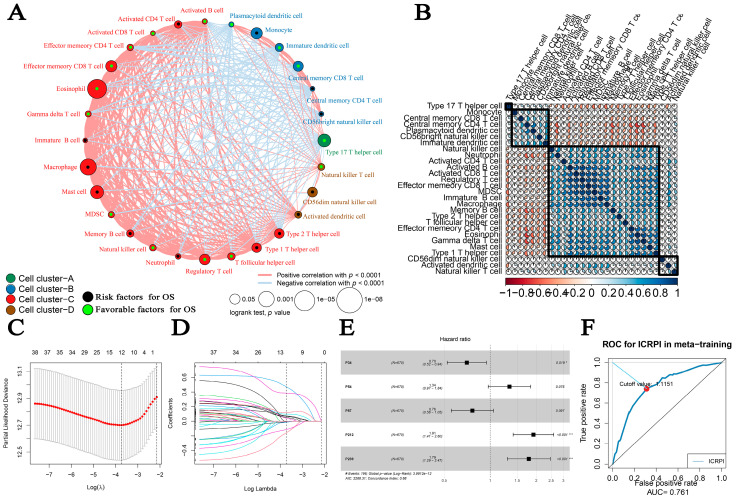
Survival landscape of the 28 immune cell types and immune-cell-related prognostic index (ICRPI) construction. (**A**) Cellular interaction and survival landscape of the 28 immune cell types. (**B**) The relationship among the 28 immune cell types. (**C**,**D**) Plot of partial likelihood deviance for the 12 immune cell pairs (ICPs) associated with survival in the training set. (**E**) Forest plot for the Hazard Ratios (HRs) of ICPs used for ICRPI construction. (**F**) Time-dependent ROC curve for ICRPI in the meta-training cohort at 5 years.

The flow chart of the entire process in this research study was shown in Figure 1. Twenty-eight immune cell types were collected and contained in this study in total. Then, ssGSEA was used for the immune infiltration-level estimation of the 28 immune cell types. Multivariate Cox analyses were obtained to measure the prognostic value of the 28 types of immune cells (Table S2). Figure 2A described a comprehensive landscape of immune cell interactions, cell lineages, and their roles on the overall survival (OS) of HCC patients. These immune cell types were separated into four clusters via the “hclust” method (Figure 2B). The relationship between immune cells is complex. More than half of them showed strong positive correlations with each other. Some immune cell types showed a strong negative correlation with others, such as gamma delta T cell and central memory CD4 T cell (Figure 2A). Moreover, some immune cell types had positive correlations with others, such as gamma delta T cell with eosinophil (Figure 2A). The result indicated that these immune cells played their roles in the tumor immune microenvironment by mutual promotion or antagonism. Furthermore, these immune cell types also showed inconsistent roles in the prognosis of HCC patients. We defined several types of immune cells, including eosinophil (HR = 0.008, *p* = 0.001), type 17 T helper cell (HR = 0.607, *p* = 0.016), and effector memory CD8 T cell (HR = 0.053, *p* = 0.043) as favorable factors for the OS of HCC patients. In contrast, some other immune cell types containing macrophages (HR = 65.16, *p* = 0.004), mast cells (HR = 12.033, *p* = 0.018), and monocytes (HR = 30.915, *p* = 0.041) were defined as elements at risk of HCC’s OS. As we mentioned in the Methods section, 374 immune cell pairs (ICPs) were generated with the use of the proportion of each immune cell type (Table S3). By performing a log-rank test, 38 ICPs were screened via the entire meta-cohort (Table S4), which were further contained in the LASSO algorithm (Table S4). In total, 12 ICPs were screened (Figure 2C,D) and 5 were generated by 10 immune cell types and were picked out by a multivariate Cox regression analysis and further used for ICRPI construction (Figure 2E and Figure S2A; Table S5). The AUC value of the time-dependent ROC curve at 5 years was 0.761 (Figure 2F), the grouping value of ICPRI was identified as 1.1151, and HCCs were subsequently classified into ICRPI high- and low-risk subgroups.

### 3.2. ICRPI Was Associated with Survival and Clinical Features of HCC

**Figure 3:**
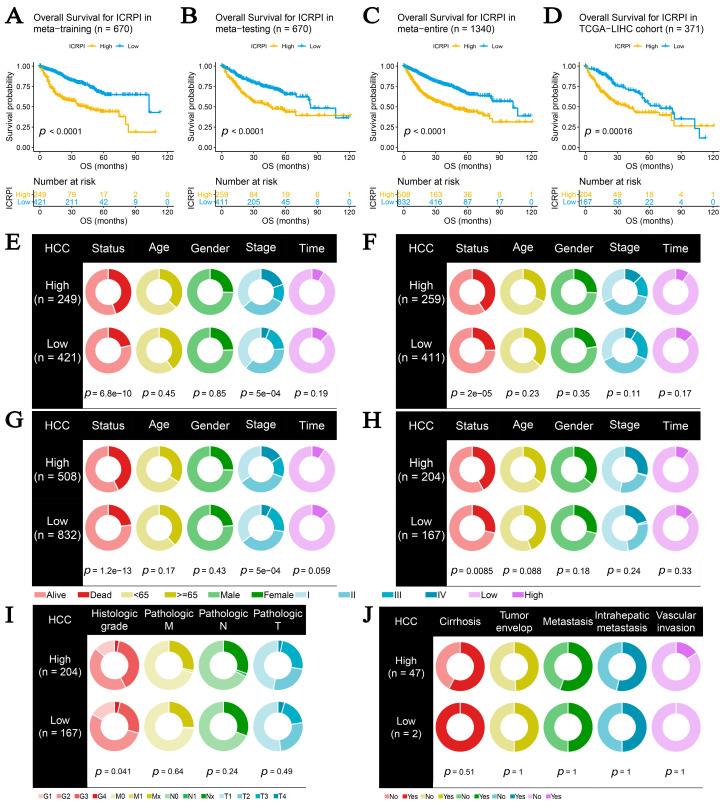
Survival, clinical, and pathological difference across ICRPI high-risk and ICRPI low-risk groups. (**A**) Overall survival curve for ICPRI in the meta-training cohort. (**B**) Overall survival curve for ICPRI in the meta-testing cohort. (**C**) Overall survival curve for ICPRI in the entire meta-cohort. (**D**) Overall survival curve for ICPRI in the TCHA-LIHC’s data. The differences in clinical features (living status, age, gender, TNM stage, and survival time) across ICRPI risk groups via the meta-training cohort (**E**), meta-testing cohort (**F**), entire meta-cohort (**G**), and TCGA-LIHC cohort (**H**). The relation of ICRPI to the pathological features (pathologic M, pathologic N, and pathologic T) via TCGA-LIHC cohort (**I**). The relationship of ICRPI to the pathological features (metastasis, intrahepatic metastasis, and vascular invasion) via cohort GSE45114 (**J**).

**Figure 4:**
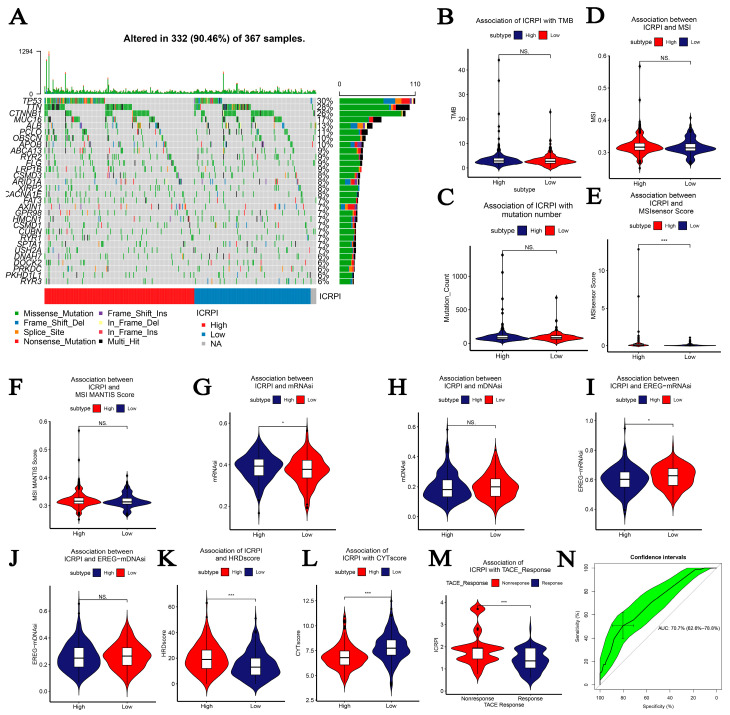
Association between ICRPI and genomic correlations and other highly trustworthy indices via TCGA-LIHC data. (**A**) The oncoplot of the top 30 mutated genes that were associated with ICRPI. (**B**) Association between ICRPI and TMB. (**C**) Association between TCPRI and mutation number. (**D**) MSI. (**E**) MSI sensor score. (**F**) MSI MANTIS score. (**G**) mRNAsi. (**H**) mDNAsi. (**I**) EREG-mRNAsi. (**J**) EREG-mDNAsi. (**K**) HRD score. (**L**) CYT score. (**M**) TACE response. (**N**) ROC curve to explore the role of ICRPI in TACE response prediction. NS no significance, * *p* < 0.05, *** *p* < 0.001.

Based on the training cohort (*n* = 670), HCC patients in the ICRPI low-risk group (*n* = 421) showed better OS compared to those of the ICRPI high-risk group (*n* = 249), as described in Figure 3A (*p* < 0.001). We obtained a similar conclusion via the meta-testing cohort (*n* of high-risk set = 259, *n* of low-risk set = 411, Figure 3B), entire meta-cohort (*n* of high-risk set = 508, *n* of low-risk set = 832, Figure 3C), and TCGA-LIHC cohort (*n* of high-risk set = 204, *n* of low-risk set = 167, Figure 3D). The ICRPI distribution of HCC patients in the four cohorts is shown in Figure S1B–I. In total, HCC patients in the ICRPI high-risk group were more prone to death, which indicated that ICRPI was a prognostic index for HCC (Figure S2B–I). We further explored the clinical difference among the ICRPI risk groups (Table S6). In the meta-training dataset, patients classified into the ICRPI high-risk set showed higher stages (*p* < 0.001) than the patients in the low-risk subgroup (Figure 3E). Furthermore, HCC patients in the ICRPI high-risk group might have had worse survival states compared with the ICRPI low-risk group (*p* < 0.001, Figure 3E), which concluded that the ICRPI could act as a prognosis stratification tool. The meta-testing cohort (Figure 3F), entire meta-cohort (Figure 3G), and TCGA-LIHC cohort (Figure 3H) were used to validate the clinical difference among the ICRPI risk groups, and the result is consistent with those shown in Figure 3E. These included no difference in age, gender, and survival time between the high-risk patient group and low-risk patient group, suggested by all data cohorts (Figure 3E–H). We also explored the relationship of ICRPI to several pathological features. As Figure 3I shows, patients classified into the ICRPI high-risk group showed higher histologic grade (*p* = 0.041) than the patients in the low-risk subgroup via cohort TCGA-LIHC. There were no significant relationships between ICRPI and pathologic M, pathologic N, and pathologic T (Figure 3I). By using cohort GSE45114, we also explored the relationship of ICRPI to metastasis, intrahepatic metastasis, and vascular invasion. As Figure 3J shows, it seems that HCC patients classified into the ICRPI high-risk group were more likely to metastasize, intrahepatic metastasize, and vascular invade, without significance. Higher tumor mutation burden (TMB), as well as higher somatic mutation rates, was associated with greater anti-cancer immunity. Figure 4A shows the mutation landscape of the top 30 high-frequency mutated genes in HCC patients via TCGA data. The ICRPI high-risk group showed a higher mutation rate (Figure 4A). There was a trend that ICRPI high-risk group contained higher TMB compared to the ICRPI low-risk group (Figure 4B), in addition to the number of mutated genes (Figure 4C), without any significance.

### 3.3. The Correlation of ICRPI with Several Highly Trustworthy Indices

MSI has been identified as a meaningful marker for cancer diagnosis and treatment across a set of cancer types. A tendency had been proved that HCCs in the ICRPI high-risk set showed a greater MSI (Figure 4D, no significance), MSIsensor score (Figure 4E, *p* < 0.001), and MSI MANTIS score (Figure 4F, no significance) than those in the ICRPI low-risk group. mRNAsi was defined as a novel predictor associated with stem-like indices and tumor prognosis. HCCs in the ICRPI high-risk subgroup showed higher mRNAsi (Figure 4G, *p* < 0.05) and lower EREG-mRNAsi (Figure 4I, *p* < 0.05) compared to the ICRPI low-risk group. A further analysis concluded that there were no statistical differences (mDNAsi (Figure 4H) and EREG-mDNAsi (Figure 4J)) among the ICRPI risk groups. The HRD score represents distinct types of genomics scar and chromosomal instability caused by deoxyribonucleic acid repair deficiency, regarded as a powerful biomarker of a given cancer. Samples in the ICRPI low-risk group showed lower HRD scores compared to those in the ICRPI high-risk group (*p* < 0.001, Figure 4K). The cytolytic activity (CYT) score is a new index of cancer immunity calculated from the mRNA expression levels of GZMA and PRF1; we concluded that the HCC patients with higher ICRPI had lower CYT scores compared to those observed in lower ICRPI patients (*p* < 0.01, Figure 4L). The details about these indices are shown in Table S7.

### 3.4. The Role of ICRPI in TACE Treatment Response

Transcatheter arterial chemoembolization (TACE) treatments have been widely used for unresectable live cancer treatments. Currently, this treatment method is the preferred therapy for patients with advanced liver cancer. By using GSE104580, we found that HCC patients that had no response to TACE treatments showed higher ICRPI levels comparing with HCC patients that were responsive to TACE treatments (Figure 4M, *p* < 0.001). Moreover, the ICRPI was concluded to have predictive values in HCC responses relative to TACE, with an AUC value of 0.707 (Figure 4N), which might be a biomarker for predicting HCC responses to TACE.

### 3.5. Association between ICRPI and Immune Related Features

**Figure 5:**
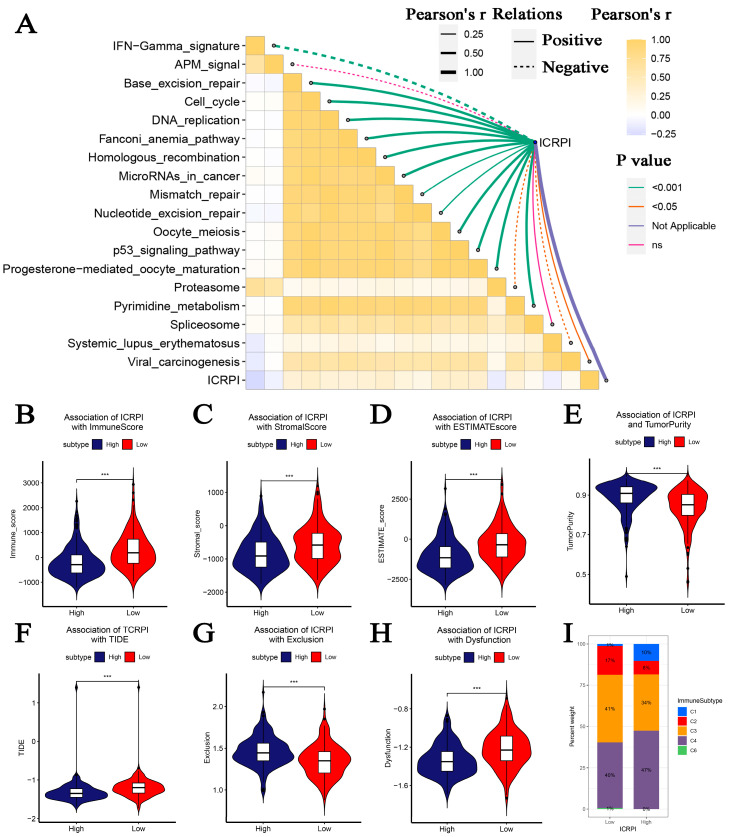
Correlation of ICRPI with immune-related features. (**A**) Association between ICRPI and immunotherapy response related pathways. (**B**) Association between ICRPI and immune score. (**C**) Association between ICRPI and stromal score. (**D**) Association between ICRPI and ESTIMATE score. (**E**) Association between ICRPI and tumor purity. (**F**) Association between ICRPI and TIDE. (**G**) Association between ICRPI and exclusion. (**H**) Association between ICRPI and dysfunction. (**I**) Association between ICRPI and immune subtypes. NS no significance, *** *p* < 0.001.

**Figure 6:**
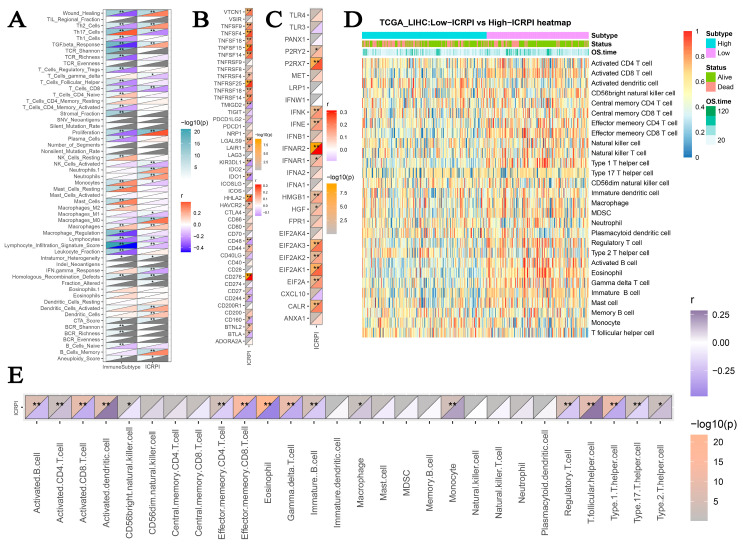
Immune landscape of ICRPI in HCC. (**A**) Association between ICRPI and 56 molecular signatures. (**B**) Association between ICRPI and ICPs. (**C**) Association between ICRPI and ICD modulators. (**D**) Heatmap showed the immune infiltration levels of the 28 immune cell types defined by ssGSEA. (**E**) Association between ICRPI and 28 immune cell types defined by ssGSEA. * *p* < 0.05, ** *p* < 0.01.

Subsequently, the associations among ICRPI and immunotherapy-related pathways were explored. Figure 5A indicated that the ICRPI was positively related to base excision repair (*p* < 0.001), cell cycle (*p* < 0.001), DNA replication (*p* < 0.001), Fanconi anemia pathway (*p* < 0.001), homologous recombination (*p* < 0.001), microRNAs in cancer (*p* < 0.001), mismatch repair (*p* < 0.001), nucleotide excision repair (*p* < 0.001), oocyte meiosis (*p* <0.001), p53 signaling pathway (*p* < 0.001), progesterone-mediated oocyte maturation (*p* < 0.001), pyrimidine metabolism (*p* < 0.001), and viral carcinogenesis (*p* < 0.05). The ICRPI also showed significantly negative associations with IFN-Gamma signatures (*p* < 0.001), proteasome (*p* < 0.05), and systemic lupus erythematosus (*p* < 0.05). Furthermore, patients classified into the ICRPI low-risk group showed higher levels in the immune score (*p* < 0.001), stromal score (*p* < 0.001), and ESTIMATE score (*p* < 0.001) compared to the ICRPI high-risk group (Figure 5B–D). Moreover, HCCs split into the ICRPI high-risk subgroup showed greater tumor purity comparing with ICRPI low-risk group patients (Figure 5E, *p* < 0.001). Higher TIDE scores indicated that patients were less likely to benefit from ICI treatment. Figure 5F–H suggested a conclusion that HCCs in the ICRPI high-risk subgroup probably benefit from ICI treatments more easily (*p* < 0.001). Patients in the ICRPI risk groups mainly overlapped with C3 and C4 (Figure 5I). Meanwhile, 17% of HCCs in the ICRPI low-risk subgroup were classified into C2, and more HCCs in ICRPI low-risk subgroup were categorized into C3; meanwhile, more HCCs in the ICRPI high-risk group were categorized into C4 (Figure 5G). The 56 molecular were collected from previous studies. Further analyses indicated that the ICRPI was positively associated with wound healing, Th2 cells, TGF beta response, proliferation, neutrophils, macrophages M0, dendritic cells activated, dendritic cells, and B cells memory (Figure 6A). In addition, ICRPI was negatively correlated with Th17 cells, T cells gamma delta, T cells CD8, activated NK cells, monocytes, macrophages M1, lymphocytes, lymphocyte infiltration signature score, and IFN gamma response (Figure 6A). Furthermore, the correlation between ICRPI and immune modulators including ICPs and ICD modulators was explored, Figure 6B showed the correlation of ICRPI with ICPs, and the ICRPI was positively associated with VTCN1, TNFSF9, TNFSF4, TNFSF18, TNFSF15, TNFSF14, TNFRSF25, TNFRSF18, TNFRSF14, LGALS9, LAIR1, HHLA2, HAVCR2, CD44, CD276, and BTNL2. Moreover, the ICRPI was negatively related with TMIGD2, KIR3DL1, IDO1, CD48, CD244, CD160, and BTLA (Figure 6B). Moreover, the ICRPI was positively correlated with P2RY2, P2RX7, IFNK, IFNE, IFNAR2, IFNAR1, HMGB1, HGF, EIF2AK3, EIF2AK2, EIF2AK1, EIF2A, and CALR (Figure 6C). Then, we explored the association between ICRPI and 28 immune cell types (Figure 6D,E). The ICRPI was positively correlated with six immune cell types, including activated CD4 T cell, activated dendritic cell, macrophage, monocyte, T follicular helper cell, and Type 2 T helper cell. The ICRPI is negatively related to 11 immune cell types (eosinophil, Type 1 T helper cell, Gamma delta T cell, etc.), which is coincident with Figure 6A.

### 3.6. The ICRPI Could Predict the Immunotherapeutic Benefit

**Figure 7:**
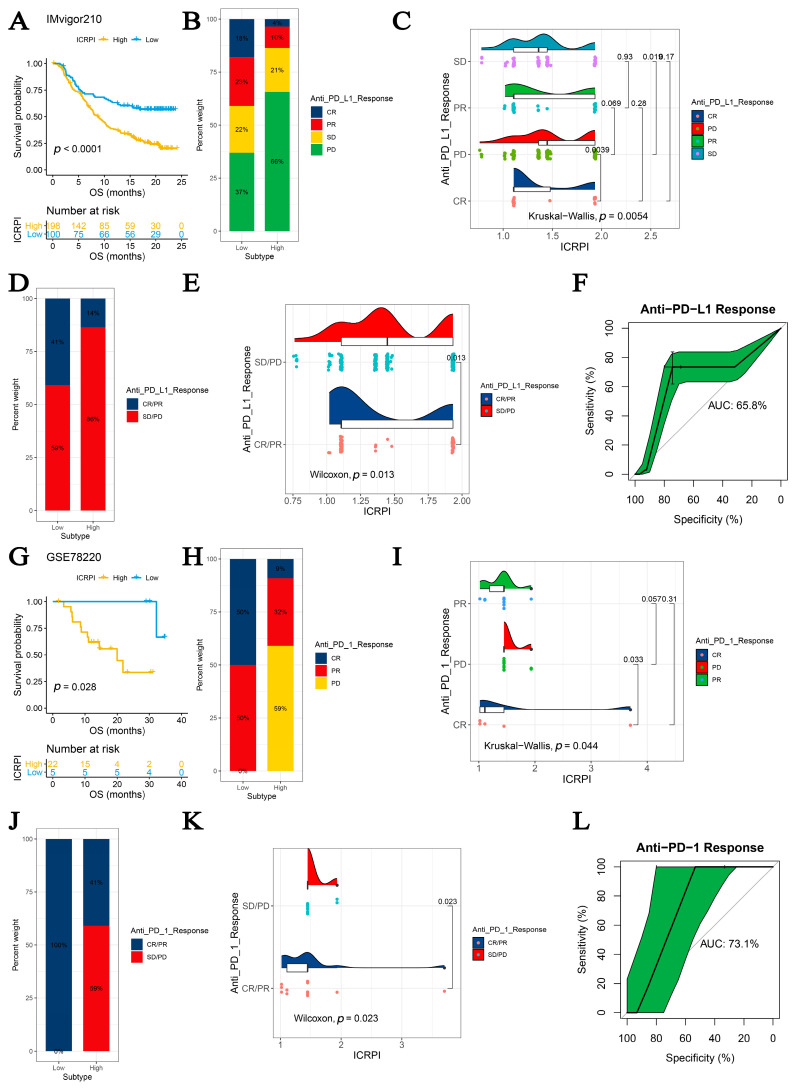
ICRPI is a prognostic biomarker and predicts immunotherapeutic benefits. (**A**) Kaplan–Meier curves for patients with high (*n* = 198) and low (*n* = 100) ICRPI in the IMvigor210 cohort. (**B**) Rate of clinical response (complete response (CR)/ partial response (PR) and stable disease (SD)/progressive disease (PD)) relative to anti-PD-L1 immunotherapy in high or low ICRPI groups in the IMvigor210 cohort. (**C**) Distribution of ICRPI in groups with different anti-PD-L1 clinical response statuses. (**D**) Rate of clinical response (CR, PR, SD, and PD) relative to anti-PD-L1 immunotherapy in high or low ICRPI groups in the IMvigor210 cohort. (**E**) Distribution of ICRPI in groups with different anti-PD-L1 clinical response statuses. (**F**) ROC curve measuring the predictive value of the ICRPI. (**G**) Kaplan–Meier curves for patients with high (*n* = 22) and low (*n* = 5) ICRPI in the GSE78220 cohort. (**H**) Rate of clinical response (CR/PR and SD/PD) relative to anti-PD-1 immunotherapy in high or low ICRPI groups in the GSE78220 cohort. (**I**) Distribution of ICRPI in groups with different anti-PD-1 clinical response statuses. (**J**) Rate of clinical response (CR, PR, SD, and PD) to anti-PD-1 immunotherapy in high or low ICRPI groups in the GSE78220 cohort. (**K**) Distribution of ICRPI in groups with different anti-PD-1 clinical response statuses. (**L**) ROC curve measuring the predictive value of the ICRPI.

Immunotherapies such as PD-L1 and PD-1 blockade unquestionably emerged great advances in tumor treatments. In IMvigor210, HCC patients in the ICRPI low-risk subgroup (*n* = 100) had longer survival (*p* < 0.001, Figure 7A) compared to HCCs classified into the ICRPI high-risk subgroup (*n* = 198). The predictive value of the ICRPI relative to anti-PD-L1 immune therapy was further confirmed (Figure 7B–E). Patients divided into the ICRPI low-risk subgroup could benefit from anti-PD-L1 treatments better (Figure 7B, 7D), which was validated by the Kruskal–Wallis test (*p* = 0.0054) and Wilcoxon test (*p* = 0.013), as Figure 7C,E suggested. HCCs with SD states for anti-PD-L1 responses showed the highest ICRPI levels among all anti-PD-L1 response states (Figure 7C). ICRPI was indicated to be a predictive biomarker relative to anti-PD-L1 immunotherapy benefits (AUC: 0.658, Figure 7F). Furthermore, with the use of the GSE78220 dataset, the role of ICRPI in anti-PD-1 treatment responses was then explored. Patients divided into the ICRPI low-risk subgroup had better survival by comparison with the ICRPI low-risk subgroup (*p* = 0.028, Figure 7G). Similarly, patients in the ICRPI low-risk group could respond to anti-PD-1 immunotherapy better by comparisons with those classified into the ICRPI high-risk subgroup (Figure 7H,J), concluded by the Kruskal–Wallis test (*p* = 0.044) and Wilcoxon test (*p* = 0.023), as Figure 7I,K suggested. The HCCs with CR states for anti-PD-1 responses had the lowest ICRPI scores among all groups (Figure 7I). The ICRPI was then concluded to be a suitable predication application for anti-PD-1 therapy benefits (AUC: 0.731, Figure 7L). Taken together, the results obviously concluded that ICRPI was associated with anti-PD-L1/PD-1 immune treatment responses, which might act upon the prediction of responses to immunotherapy.

### 3.7. Predictive Value Comparison of ICRPI with Several Molecular Signatures

**Figure 8:**
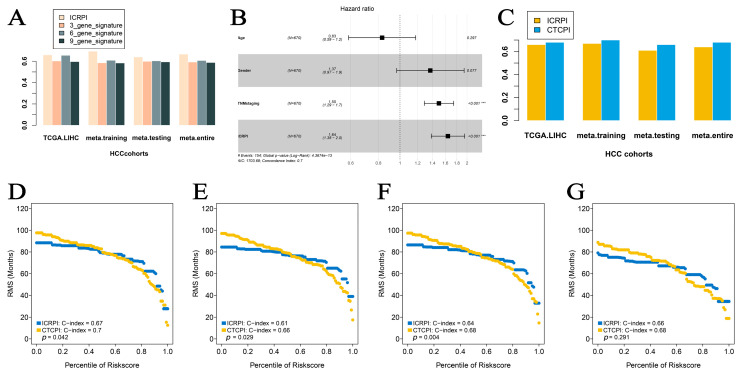
Construction of a Composite ICRPI and clinical prognostic index (ICPI). (**A**) C-index comparison between ICRPI, 3-gene signature, 6-gene signature, and 9-gene signature. (**B**) Forest plot for the Hazard Ratios (HRs) of high vs. low ICRPI risk groups via meta-training cohort. (**C**) C-index comparison between ICRPI and ICPI. Restricted mean survival (RMS) curves for continuous ICRPI and CTCPI in meta-training cohort (**D**), meta-testing cohort (**E**), entire meta-cohort (**F**), and TCGA-LIHC cohort (**G**).

To determine whether the ICRPI was better than previous prognostic signatures, three multiple gene signatures were collected and included in the present study. As shown in Figure 8A, the ICRPI showed the best prognosis prediction potential compared to the three-gene signature, six-gene signature, and nine-gene signature in the TCGA-LIHC cohort, meta-training cohort, meta-testing cohort, and entire meta-cohort.

### 3.8. Construction of ICPI and Its Prognostic Role

To maximize the application of the ICRPI in the prognosis prediction of HCC patients, we immediately contained the ICRPI and several essential clinical factors (age, gender, and TNMstaging) in the multivariable Cox analysis via the meta-training cohort (Figure 8B; Table S8). TNMstaging (HR = 1.50, *p* < 0.001) was then screened and generated with ICRPI (HR = 1.64, *p* < 0.001) to construct ICPI. The prognostic value of age was then validated by the meta-testing cohort (Figure S3A), entire meta-cohort (Figure S3B), and TCGA cohort (Figure S3C). Based on the results of the Cox result, the ICPI of HCC patients was defined as TNMstage × 0.404 + ICRPI × 0.497. The result was shown in Figure 8C, the prediction potential of ICRPI had been significantly improved by constructing ICPI in all the four data cohorts (Figure 8C) and validated by RMS curves in the meta-training cohort (mean C-index: ICPI: 0.70, ICRPI: 0.67, *p* = 0.042, Figure 8D), meta-testing cohort (mean C-index: ICPI: 0.66, ICRPI: 0.61, *p* = 0.029, Figure 8E), entire meta-cohort (mean C-index: ICPI: 0.68, ICRPI: 0.64, *p* = 0.004, Figure 8F), and TCGA-LIHC cohort (mean C-index: ICPI: 0.68, ICRPI: 0.66, *p* = 0.291, Figure 8G).

### 3.9. Novel Candidate Drugs Treating HCC

**Figure 9:**
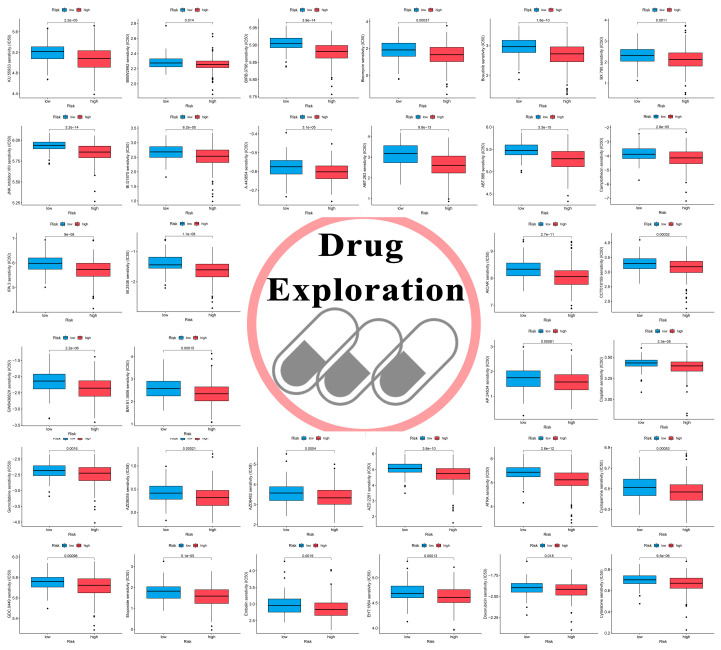
Drug sensitivity exploring to treat HCC patients classified into the ICRPI high-risk group. In total, 32 potential drugs were identified. Drugs with *p* < 0.05 were considered significant.

Then, we attempted to identify some novel candidate drugs for HCC treatments. Drugs from the GDSC database including the therapeutic ability for cancers were contained for this analysis. As the result indicated, HCC patients classified into the ICRPI high-risk subgroup were more susceptible to 57 medicine types (Figure 9 and Figure S4). Drugs such as KU.55933 (*p* = 2.2 × 10^−5^), BIRB.0796 (*p* = 3.9 × 10^−14^), and Bosutinib (*p* = 1.8 × 10^−10^) might be effective treatments for HCCs. The result indicated that ICRPI could predict increased sensitivity towards these therapeutic drugs in HCC patients.

## 4. Discussion

As major components in tumor immune microenvironments, immune cells have been proved to be correlated with the prognosis of cancers, which could also accelerate tumor initiation and progression [14,15,16]. More recently, studies have shown that the immunotherapy efficacy of HCC patients could be impacted by immune cells. A study contributed by Geh et al. concluded that neutrophils could act as potential therapeutic targets in HCC [49]. Within the tumor, macrophages could affect the development and progression of HCC. Xu et al. discussed the possible approaches for tumor-associated macrophage (TAMs) therapy as potential targets for HCC treatment [50]. T cell is the most numerous type with complex functions in lymphocytes [51]. T cells have been proved as essential effectors in anti-tumor immunity. Zhang et al. screened a tumor-infiltrating immune-cell-associated lncRNA signature, which could predict the outcomes of tumor immunotherapy [52]. These studies proved the importance of immune cells in survival, anti-tumor immunity, and therapy response in HCC. However, none of them considered the integrity and comprehensive interaction of immune cell subpopulations. Since there were a set of immune cell subpopulations, providing a landscape of these immune cell subpopulations in HCC was urgently need. In total, 28 immune cell types were included in the present study, the infiltration level of which were evaluated for over 1300 samples from public databases by applying the ssGSEA method. The ssGSEA algorithm scored the individual samples independently without considering other samples in the gene expression cohorts, which could overcome calculation errors caused by multiple platforms of cohorts. Then, the immune cell pair (ICP) method was realized to measure the interactions within immunocyte subpopulations and to further construct an immune-cell-related prognostic index (ICRPI). Moreover, the cell pair algorithm only involves pairwise comparisons within the cell infiltration level cohort of a sample, which allowed us to use samples from multiple platforms. The impact of ICRPI on survival was then measured. A subsequent analysis concluded that HCC patients with a lower ICRPI were more likely to show better survival, which indicated that the ICRPI was a risk indicator for the survival and prognosis of HCC patients. The clinical difference among ICRPI risk groups was also explored, and HCCs categorized into ICRPI low-risk subgroups were less likely die and proceeded into advance stage HCC. The 5-year survival rate of advance HCC dropped to 3%, which is consistent with the present study.

mRNAsi is a novel predictor associated with stem-like indices and tumor prognosis. A study by Mai et al. proved that HCCs with higher mRNAsi scores had significantly worse overall survival based on an HCC matrix of more than 1000 HCC patients [52]. In this paper, we concluded the ICRPI was positively associated with mRNAsi, acting as a risk factor for the survival of HCC patients, which was consistent with Mai et al [53]. HRD had been used as a biomarker for therapy decision making [54]. As a recent study reported, the HRD score can also predict responses to neoadjuvant chemotherapy in some cancer types, such as triple-negative breast cancer [55]. In HCC, Knijnenburg et al. concluded that HRD scores were often associated with shorter survival. Our result concluded that ICRPI was positively associated with the HRD score, which further suggested that HCCs with higher HRD scores had worse survival, which is consistent with a previous study [56]. Zhang et al. proved that the CYT score was a prognostic marker in HCC [23]. The present study concluded that ICRPI was negatively associated with the CYT score, which also suggested that the CYT score was a favorable factor for the survival of HCC. The TACE treatment was the major treatment for advanced HCC. In the present study, we also explored that ICRPI could act as a predictive marker for the TACE treatments for HCC, which indicated that ICRPI might act on the selection of treatment methods for HCC.

Then, we attempted to characterize the immune landscape across the ICRPI risk groups. The ICRPI might regulate some immune-related features. Specifically, Figure 5A indicates that the ICRPI is positively related to base excision repair, DNA replication, cell cycle, fanconi anemia pathway, microRNAs in cancer, homologous recombination, mismatch repair, pyrimidine metabolism, oocyte meiosis, nucleotide excision repair, p53 signaling pathway, progesterone-mediated oocyte maturation, and viral carcinogenesis. The ICRPI also showed significantly negative associations with IFN-Gamma signatures, proteasome, and systemic lupus erythematosus. These results proved that the ICRPI might effectively influence the HCC immune microenvironment via complex immune-related pathways. Furthermore, the present study concluded that ICRPI was negatively correlated with immune, stromal, and ESTIMATE scores, resulting in the lower tumor purity of HCCs with lower ICRPI scores. In addition, ICRPI was associated with some ICPs, including VTCN1, TNFSF9, TNFSF4, TNFSF18, TNFSF15, TNFSF14, TNFRSF25, TNFRSF18, TNFRSF14, LGALS9, LAIR1, HHLA2, HAVCR2, CD44, CD276, BTNL2, TMIGD2, KIR3DL1, IDO1, CD48, CD244, CD160, and BTLA, indicating that ICRPI could be an effective indicator for immune checkpoint blockage (ICB) therapy. A higher TIDE score indicated that patients were less likely to profit by ICI treatment. This study concluded that HCC patients classified into the ICRPI high-risk subgroup might profit by ICI treatments better.

Tumor immunotherapy is a novel therapy option for controlling and eliminating tumors by restarting and maintaining the tumor immune cycle and restoring the normal anti-tumor immune response [57]. Because of its excellent curative effect and innovation, it was rated as the most important scientific breakthrough of the year by *Science* in 2013. Thus, the role of the ICRPI in immunotherapy must be explored in this research area. Further integrated analyses cleared up the point about the association between ICRPI and response to anti-PD-L1/PD-1 immune therapy, which might help improve the predictive strategy for immunotherapy.

Except immunotherapy, drug therapy, especially chemotherapy, is also the major therapy strategies for HCC. Then, we attempted to identify some novel candidate drugs for HCC treatment. Drugs from the GDSC database, including the therapeutic ability for cancers, were contained for this analysis. HCC patients classified into the ICRPI low-risk group were less sensitive to 57 drugs. Drugs such as KU.55933, BIRB.0796, and Bosutinib might be effective treatments for HCCs, indicating that ICRPI could predict increased sensitivity towards these therapeutic drugs in HCC patients.

To maximize the application of the ICRPI in the prognosis prediction of HCC patients, we then constructed an ICPI by considering both ICRPI and several clinical features. The C-index improved from 0.67 to 0.70, which truly improved the prediction of survival of HCC patients.

The present study also has certain limitations. Although we collected and used as many public HCC cohorts as possible, we lacked our own data for external verification. In future, we will collect HCC patients from our hospital and further validate the ICRPI. Furthermore, it seemed that HCC patients divided into the ICRPI high-risk group were more likely to metastasize, intrahepatic metastasize, and vascular invade, without significance. Perhaps because of the small size of cohort GSE45114, in the near future, we will also validate the relationship of ICRPI to pathological features using our own data. Moreover, aside from data size, data quality must also be considered when conducting scientific research. Although we collected the HCC data from several widely used public databases, which provided some guarantee of the data quality, the quality of the data was not clear. To minimize the impact of data quality on the deduced conclusions in the present study, we only collected HCC cohorts with a relative complete gene expression matrix, and survival and clinical information. For HCC cohorts from different platforms, we used different methods for individual normalization to ensure the correct standardized methods were used. Then, the ComBat-adjusted algorithm was chosen to merge the entire meta-cohort. Moreover, to overcome the difficulty of using multiple datasets from different platforms, we used an immune-cell-pair algorithm to construct the ICRPI. Thus, we thought the deduced conclusion in the present study must be credible. As for the influence of the quality of data on our research, these must be in-depth analyses in the future using these public data and our own data.

Taken together, the present study put forward some novel insights for increasing the survival estimation and response to treatment (TACE, immunotherapy, and drug therapy) for individual HCC patients via a comprehensive analysis of immune cell types, which might take effect on the personalized precision immunotherapy strategy of HCC over the next decades.

## 5. Conclusions

In general, we constructed and verified an immune-cell-related prognostic index (ICRPI) in the present study; it is an effective tool for predicting the prognosis of HCC and distinguishing patients that are suitable for immunotherapy. The comprehensive evaluation of the interactions of immunocytes in HCC might improve the cognition of the infiltration characteristics and functions of immune cells and guide more effective immunotherapy strategies.

## Data Availability

All database generated/analyzed for this study are included/have their accession numbers included in the article.

## Supplementary Materials

The following supporting information can be downloaded at https://www.mdpi.com/article/10.3390/cells12010202/s1, Figure S1: Power calculation to estimate the sample size in all cases including t test (A,B), one-way ANOVA (C), and correlation analysis (D). Figure S2: (A) Forest plot for the Hazard Ratios (HRs) of ICPs used for ICRPI construction. (B-I) The distribution of ICRPI in meta-training cohort (B,C), meta-testing cohort (D,G), entire meta-cohort (E,H), and TCGA-LIHC cohort (F,I). Figure S3: Forest plot for the Hazard Ratios (HRs) of high vs. low ICRPI risk groups via meta-testing cohort 1 (A), entire meta-cohort (B), and TCGA-LIHC cohort (C). Figure S4: Drug sensitivity exploring to treat HCC patients classified into ICRPI high-risk group. In total, 25 potential drugs were identified. Drugs with *p* < 0.05 were thought to be significant. Table S1: Basic information of ten included HCC cohorts. Table S2: Multivariate Cox analysis for the 22 immune cell types. Table S3: The normalized enrichment scores for the 22 immune cell types among 1487 BLCA patients. Table S4: Details about the immune cell pairs (ICPs) and survival analysis for ICPs by using the training cohort and testing cohort. Table S5: Model information about the ICRPI. Table S6: ICRPI score, clinical information, and pathological features for the 1340 LIHC patients. Table S7: The details about the LIHC patients in TCGA-LIHC cohort. Table S8: The ICPI score in the four cohorts.
