# Supplementary figures and images for: Cell Pair Algorithm-Based Immune Infiltrating Cell Signature for Improving Outcomes and Treatment Responses in Patients with Hepatocellular Carcinoma
Source: Cells. 2023 Jan 3;12(1):202. doi: 10.3390/cells12010202 (PMC9818873; doi:10.3390/cells12010202)

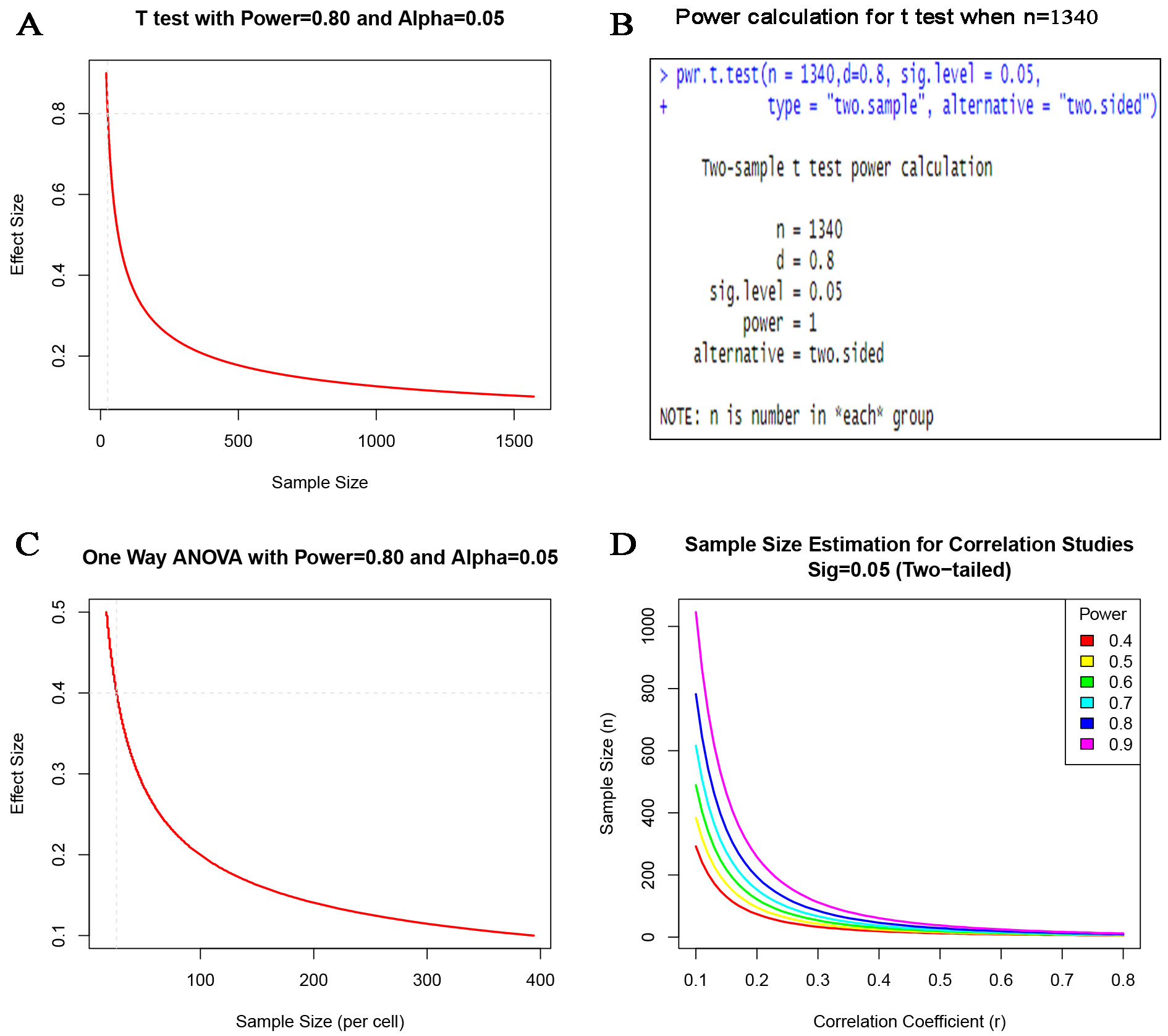

Supplement: Supplementary file 1 [file cells-12-00202-s001.zip › Figure S1.tif]

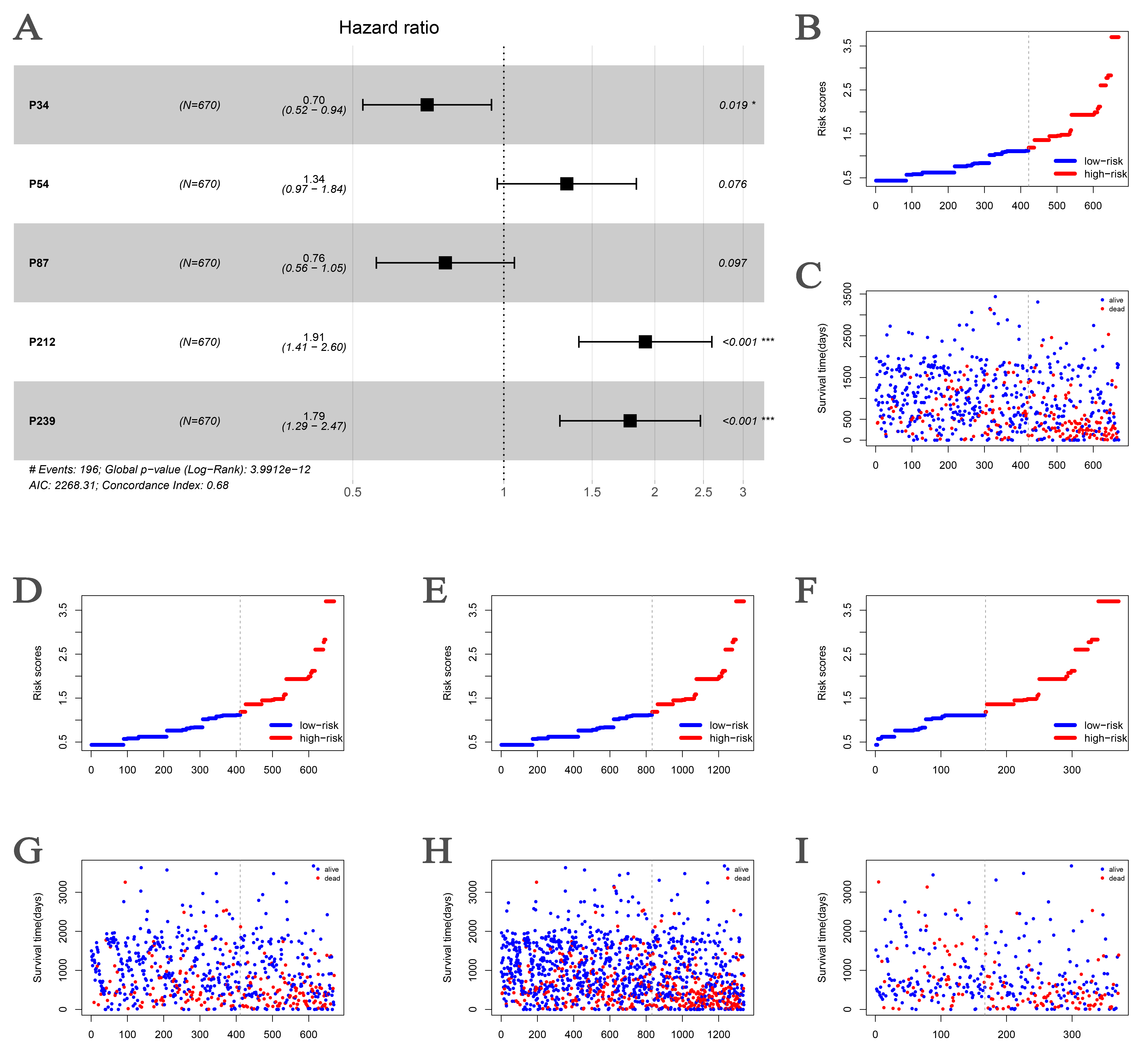

Supplement: Supplementary file 1 [file cells-12-00202-s001.zip › Figure S2.tif]

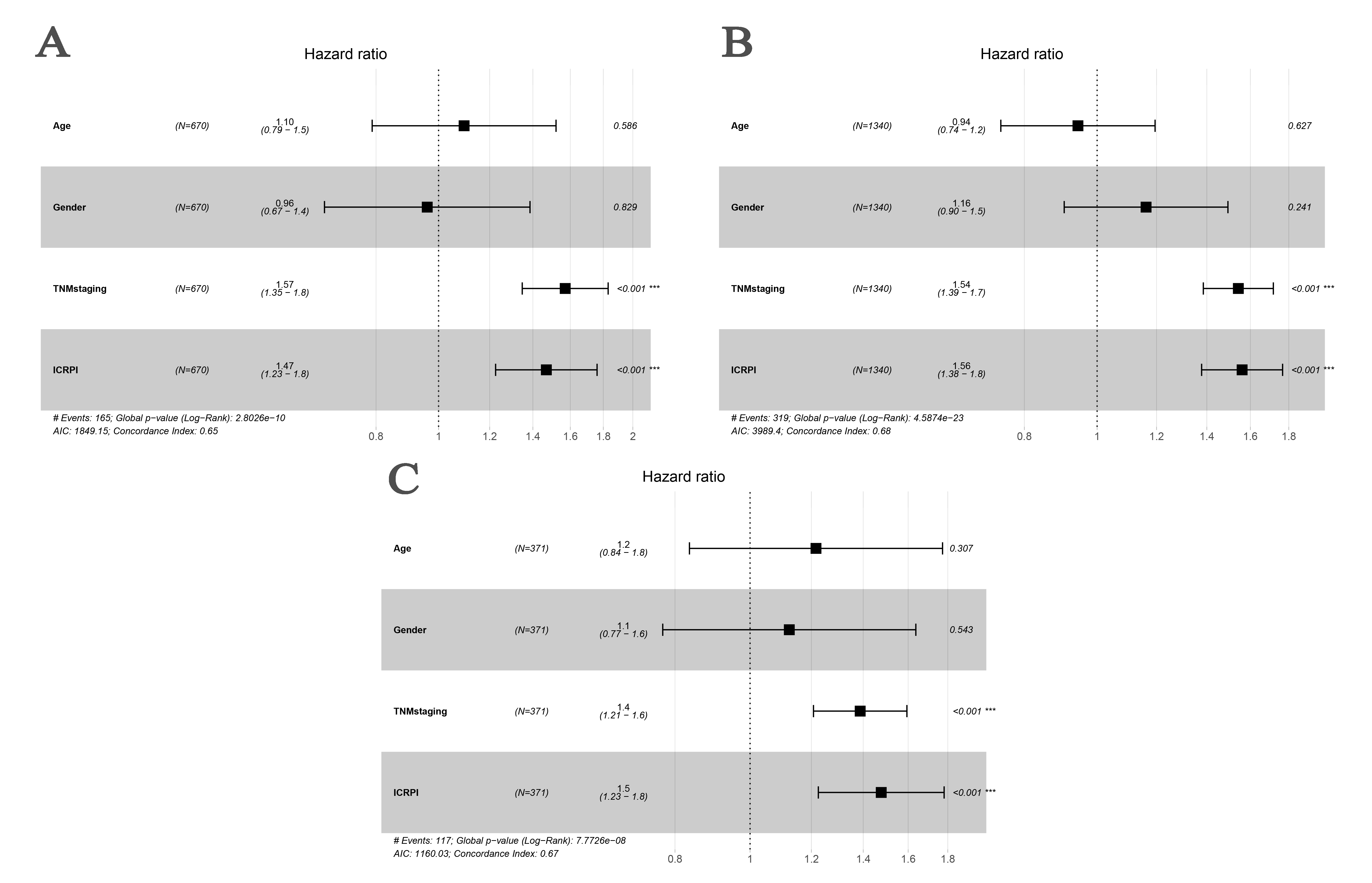

Supplement: Supplementary file 1 [file cells-12-00202-s001.zip › Figure S3.tif]

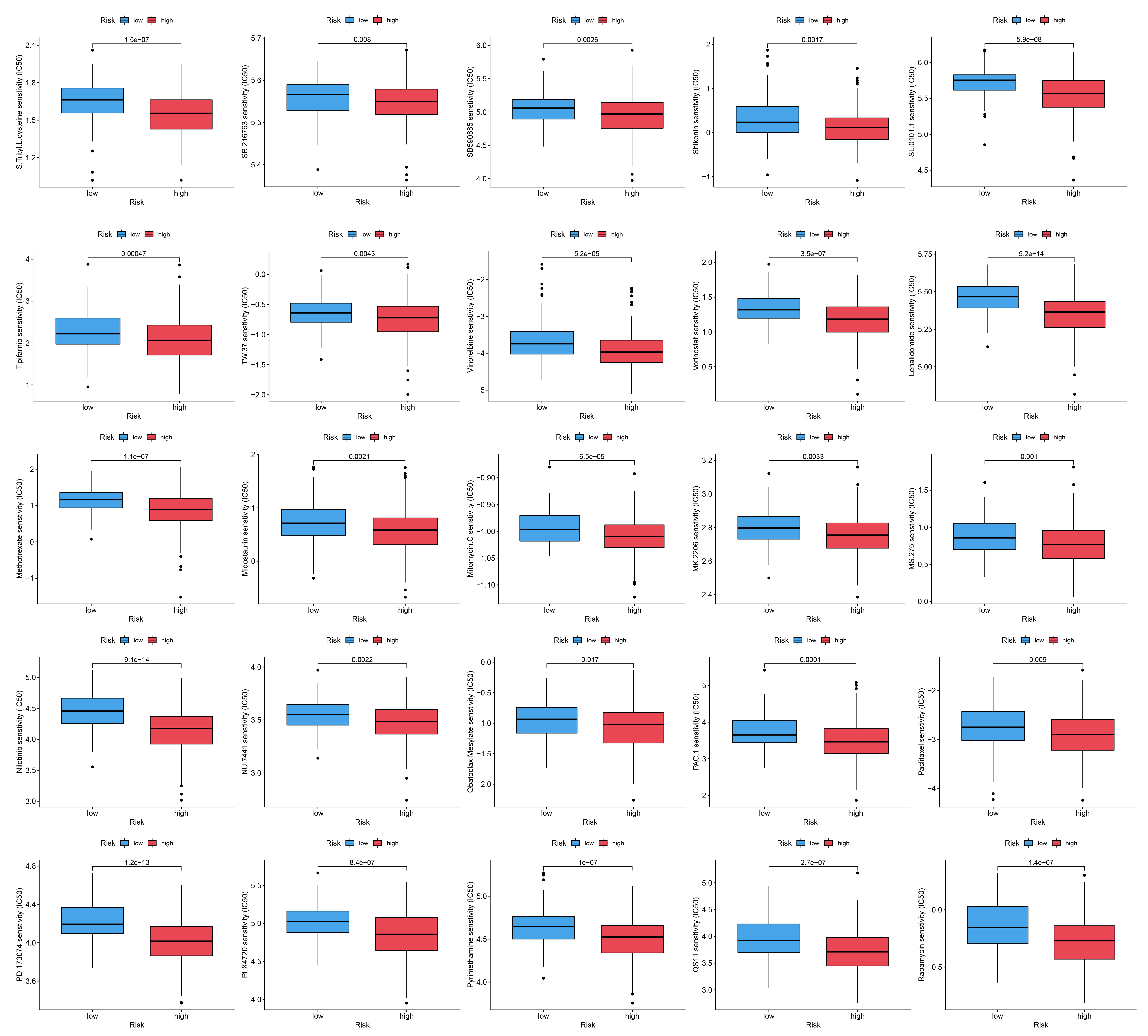

Supplement: Supplementary file 1 [file cells-12-00202-s001.zip › Figure S4.tif]
